# Phylogenetic reconstruction from transpositions

**DOI:** 10.1186/1471-2164-9-S2-S15

**Published:** 2008-09-16

**Authors:** Feng Yue, Meng Zhang, Jijun Tang

**Affiliations:** 1 Department of Computer Science and Engineering, University of South Carolina, Columbia, SC 29208, USA; 2 College of Computer Science and Technology, Jilin University, Changchun 130012, PR China

## Abstract

**Background:**

Because of the advent of high-throughput sequencing and the consequent reduction in the cost of sequencing, many organisms have been completely sequenced and most of their genes identified. It thus has become possible to represent whole genomes as ordered lists of gene identifiers and to study the rearrangement of these entities through computational means. As a result, genome rearrangement data has attracted increasing attentions from both biologists and computer scientists as a new type of data for phylogenetic analysis. The main events of genome rearrangements include inversions, transpositions and transversions. To date, GRAPPA and MGR are the most accurate methods for rearrangement phylogeny, both assuming inversion as the only event. However, due to the complexity of computing transposition distance, it is very difficult to analyze datasets when transpositions are dominant.

**Results:**

We extend GRAPPA to handle transpositions. The new method is named GRAPPA-TP, with two major extensions: a heuristic method to estimate transposition distance, and a new transposition median solver for three genomes. Although GRAPPA-TP uses a greedy approach to compute the transposition distance, it is very accurate when genomes are relatively close. The new GRAPPA-TP is available from .

**Conclusion:**

Our extensive testing using simulated datasets shows that GRAPPA-TP is very accurate in terms of ancestor genome inference and phylogenetic reconstruction. Simulation results also suggest that model match is critical in genome rearrangement analysis: it is not accurate to simulate transpositions with other events including inversions.

## Background

While phylogenetic studies in the pre-genome era primarily focused on DNA or protein sequence differences among organisms, informative comparisons can in fact be made at various organizational levels. Higher-level evolutionary events of relevance to phylogenetics include inversion, transposition, deletion, insertion and duplication. Phylogenetic analyses of whole genomes that model these types of events are proving to be extremely useful in elucidating the evolutionary relationships among organisms [1]. Since the pioneering papers of Sankoff [2], genome rearrangement data has attracted increasing attention from both biologists and computer scientists as a new type of data for phylogenetic analysis and comparative genomics.

During the past several years, computer scientists have been able to make substantial progress in genome rearrangement research. With solutions for inversion distance [3] and inversion median [4], we were able to estimate phylogenies and ancestral genomes based on inversions. The main software packages for reconstructing the inversion (or breakpoint) phylogeny are GRAPPA [5] and MGR [6]. Their basic optimization tool is an algorithm for computing the inversion (or breakpoint) median of three genomes.

Much of the research on genome rearrangement has focused on organellar genomes, such as mitochondrial [7] and chloroplast genomes [8]. GRAPPA and MGR have been applied successfully to chloroplast genomes in which inversion is the most important event. In other datasets (e.g., mitochondrial genomes), transpositions are viewed as more likely, although their relative preponderance with respect to inversions is unknown.

Existing methods can still be applied when transposition is the dominant event. For example, given genome 1, 2,⋯, *n*, a transposition acts on three indices *i*, *j*, *k *(*i *≤ *j *and *k *∉ [*i*, *j*]) resulting in a genome: 1,⋯, (*i *- 1), (*j *+ 1),⋯, *k*, *i*, (*i *+ 1),⋯, *j*, (*k *+ 1),⋯, *n*, which can also be obtained by using three inversions: one inversion acts on indices *i*, *k*, followed by one acts on indices *i*, *k *- *j *+ *i *- 1 and another one acts on *k *- *j *+ *i*, *k*. Based on the above observation, it is possible to estimate the transposition distance by inversions and use distance-based method (such as neighbor-joining) to reconstruct the phylogeny. We can also apply GRAPPA or MGR to obtain the phylogeny, using either breakpoint median solver or inversion median solver. However, since the evolutionary model is mismatched, their performance on transposition datasets is questionable, as indicated by our experimental results shown in the next section. In this paper, we introduce a new method to solve the transposition median problem and use it to infer phylogenies and ancestral genomes from datasets where transposition is the only event. The new method (GRAPPA-TP) is an extension of GRAPPA and is available free from .

### Genome rearrangements

We represent a genome as a signed ordering of *n *genes, and each gene *i *is given an orientation that is either positive, written *i*, or negative, written -*i*. Genomes can evolve through events such as inversions, transpositions and transversions, as well as other events. When transposition is the only event, the sign of each gene is irrelevant and can be ignored. Let *G *be the genome with signed ordering of 1, 2,⋯, *n*. An inversion (also called reversal in some literatures) between indices *i *and *j *(*i *≤ *j*), transforms *G *to a new genome with linear ordering

1, 2,⋯, (*i *- 1), -*j*, -(*j *- 1),⋯, -*i*, (*j *+ 1),⋯, *n*

A *transposition *on genome *G *acts on three indices *i*, *j*, *k*, with *i *≤ *j *and *k *∉ [*i*, *j*], picking up the interval *i*, (*i *+ 1),⋯, *j *and inserting it immediately after *k*. Thus genome *G *is replaced by (assume *k *> *j*):

1,⋯, (*i *- 1), (*j *+ 1),⋯, *k*, *i*, (*i *+ 1),⋯, *j*, (*k *+ 1),⋯, *n*

An *transversion *is a transposition followed by an inversion of the transposed subsequence; it is also called an *inverted transposition*.

There are additional events for multiple-chromosome genomes, such as *translocation *(the end of one chromosome is broken and attached to the end of another chromosome), *fission *(one chromosome splits and becomes two) and *fusion *(two chromosomes combine to become one).

### Distance computation

Given two genomes *G*_1 _and *G*_2_, we define the *edit distance d*(*G*_1_, *G*_2_) as the minimum number of events required to transform one genome into the other.

The *breakpoint distance *[2] is not a direct evolutionary distance measurement. A breakpoint in *G*_1 _is defined as an ordered pair of genes (*i*, *j*) such that *i *and *j *are adjacent in *G*_1 _but not in *G*_2_. The breakpoint distance is simply the number of breakpoints in *G*_1 _relative to *G*_2_.

When only inversions are allowed, the edit distance is the *inversion distance*. Hannenhalli and Pevzner [3] developed a mathematical and computational framework for signed gene-orders and provided a polynomial-time algorithm to compute the edit distance between two signed gene-orders under inversions; Bader et al. [9] later showed that this edit distance can be computed in linear time. However, computing the inversion distance is NP-hard in the unsigned case [4].

The *transposition distance *is the minimum number of transpositions needed. Computing the transposition distance is of unknown complexity and after 10 years of research, the best available method is only a 1.375-approximation [10].

Yancopoulos et al. [11] proposed a "universal" double-cut-and-join (DCJ) operation that accounts for inversions, translocations, fissions and fusions, resulting in a new genomic distance that can be computed in linear time. A DCJ operation makes a pair of cuts and proceeds to reglue cut ends, which can yield an inversion, a fission, a fusion, and a translocation. Combining two DCJ operations can create a block interchange and sometime a transposition. Although there is no direct biological evidence for DCJ operations, these operations are very attractive because it provides a unifying model for genome rearrangement [12] and it is simple to compute the DCJ distance.

### Median problem of three

The median problem on three genomes is to find a single genome that minimizes the sum of pairwise distances between itself and each of the three given genomes. This problem is computationally very hard even for the simplest breakpoint distance [13].

The *breakpoint median *problem can be transformed into a special instance of the well-studied Traveling Salesperson Problem [2], hence can be solved relatively efficient. The *inversion median *problem is to find a median genome that minimizes the summation of inversion distances on the three edges. Two exact median solvers have been proposed, all using a branch-and-bound strategy. Caprara's solver [4] is based on an extension of the breakpoint graph, while the one developed by Siepel and Moret [14] runs a direct search. Using the inversion median has dramatically improved the accuracy of genome rearrangement analysis [15]. Two heuristic methods, MGR [6] and rEvoluzer [16], are also proposed to improve the speed of inversion median, at a sacrifice of accuracy. Zhang et al. later improved Caprara's inversion median solver so that it can handle the DCJ distance [17].

### Phylogenetic reconstruction from genome rearrangements

Reconstructing phylogenies from genome rearrangement data is computationally much harder than from sequence data. For example, finding the minimum number of evolutionary events given a fixed tree can be done in linear time if the leaves are labeled with DNA or protein sequences, whereas such task for genome rearrangement data is NP hard even when the tree has only three leaves.

Methods for reconstructing trees based on genome rearrangement data include distance-based methods (for example, neighbor-joining [18]), maximum parsimony methods based on encodings [19,20], and direct optimization methods. The latter, pioneered by Sankoff and Blanchette [2] in their package BPAnalysis and improved by GRAPPA [5] and MGR, is the most accurate method. Besides returning a phylogeny, these three methods can also give an estimate of ancestral gene orders, which will have great utility for biologists interested in the process of genome rearrangement.

## Results and discussion

We examine the performance of the new GRAPPA-TP through two simulation studies: the first study is to measure the accuracy of the inferred median genome (estimated ancestor) compared to the true ancestor, using datasets of three input genomes; the second is to measure the accuracy of the inferred phylogeny compared to the true tree, using datasets of 10 genomes. All the experiments are conducted on a Linux cluster with 152 Intel Xeon CPUs, but each CPU works independently on a test task.

### Accuracy of ancestor inference for three genomes

We first examine the quality of GRAPPA-TP in inferring ancestor genomes. In our simulation study, each genome has 37 or 100 genes, spanning the range from mitochondria to chloroplast.

We create each dataset by first generating a tree with three leaves and assigning its three edges with different lengths. The length (number of events) of each edge is sampled from a uniform distribution on the set {0.5*r*,..., 1.5*r*}, where *r *is the expected number of evolutionary events (only transpositions in this study). In this experiment, we use *r *= 2 ~8, where *r *= 2 is considered easy and *r *= 8 is very difficult especially for datasets with 37 genes. The gene orders on the leaves are generated by first assigning the identity permutation 1, 2,⋯, *n *(*n *= 37 *or *100) to the root, then evolving the permutation down the tree, applying along each edge a number of transpositions equal to the assigned edge length.

Given an estimated ancestor gene order *G*_*M*_, we can use the breakpoint distance between *G*_*M *_and *G*_0 _as a measurement of how close the inferred ancestor is to the true ancestor. For each dataset, we obtain the estimated ancestors by using the following five methods: GRAPPA-TP (TP), DCJ median solver (DCJ), MGR, breakpoint median solver (BP) and inversion median solver (INV). We repeat 100 times for each setting and the averages of the results are reported.

**Figure 1:**
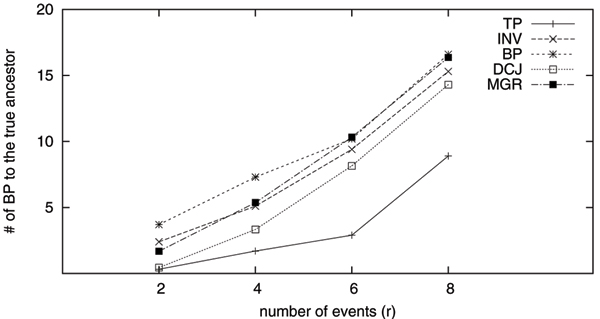
**Breakpoint distance from the inferred median to the true ancestor (37 genes)**. TP indicates the result obtained from GRAPPA-TP, INV indicates the result obtained by using the Caprara's inversion median solver, BP indicates the result obtained by using the breakpoint median solver, MGR indicates the result obtained by using MGR and DCJ indicates the result obtained by using the DCJ median solver.

**Figure 2:**
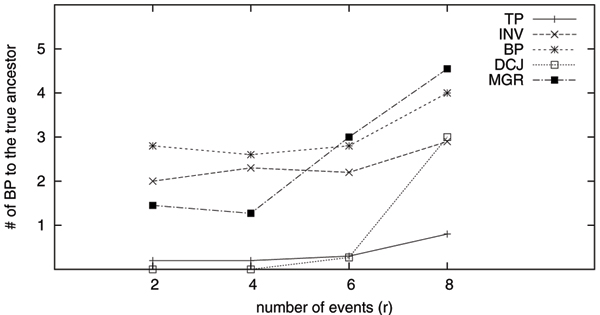
**Breakpoint distance from the inferred median to the true ancestor (100 genes)**. TP indicates the result obtained from GRAPPA-TP, INV indicates the result obtained by using the Caprara's inversion median solver, BP indicates the result obtained by using the breakpoint median solver, MGR indicates the result obtained by using MGR and DCJ indicates the result obtained by using the DCJ median solver.

Figure 1 and Figure 2 show the result. From these figures, we find that the median genomes returned by GRAPPA-TP are the closest to the true ancestors, except for the easy datasets with 100 genes and *r *= 4, where the DCJ median actually performs better. The medians returned by both breakpoint and inversion median solvers are further away from the true ancestors, a result mainly due to the usage of mismatched evolutionary models. Although DCJ and breakpoint distances are generally viewed as not so sensitive to model mismatch, our testing results directly contradict this conjecture.

**Figure 3:**
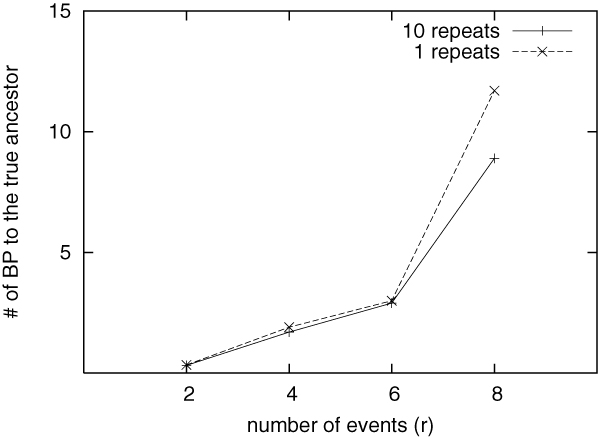
**Breakpoint distance from the inferred median to the true ancestor**. In this experiment, 1 and 10 repeats are used for the distance computation.

As indicated in the later section, GRAPPA-TP uses a simple distance estimator to conduct a randomized search, and we may need to repeat several times to obtain the smallest distance, hence the number of repeats may have impact on its performance. To assess the impact, we compare GRAPPA-TP using two numbers of repeats: 1 and 10, and report the results in Figure 3. Surprisingly this figure shows that the impact of number of repeats is very small, even when the genomes are getting distant (*r *= 6 ~8).

### Accuracy of phylogeny inference

We also test the performance of GRAPPA-TP on phylogeny analysis. We first define our measure for the accuracy of reconstructed trees. Given an inferred tree, we compare its topological accuracy by computing *false negatives *and *false positives *with respect to the true tree. For every tree there is a natural association between every edge and the bipartition on the leaf set induced by deleting the edge from the tree. Let *T *be the true tree and let *T*' be the inferred tree. An edge *e *in *T *is "missing" in *T*' if *T*' does not contain an edge defining the same bipartition; such an edge is called a *false negative *(FN). The *false negative rate *is the number of false negative edges in *T*' with respect to *T *divided by the number of internal edges in *T*. External edges (i.e. edges incident to a leaf) are not counted because these edges are trivial to recover and must present in every tree with the same set of leaves. The *false positive (FP) rate *is defined similarly, by swapping *T *and *T*'. The *Robinson-Foulds *(RF) rate is thus defined as the average of the FN and FP rates. In this study, we generate uniformly random tree by randomly picking a tree from all possible trees – there are (2*N *- 5) × (2*N *- 7) × ⋯ × 3 trees for *N *taxa. We use trees with *N *= 10 and 37 genes, which is the number of genes in mitochondrial genomes. We choose *r *= 2, 3 and 4 to vary the level of difficulty, where *r *= 4 is considered very hard for these datasets. For each combination of parameters, we generate 10 datasets and report the average results.

**Figure 4:**
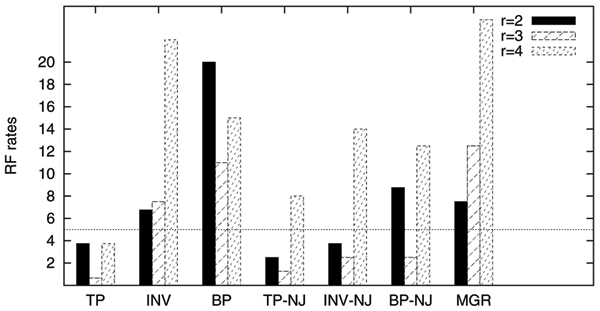
**RF errors for seven methods under different expected number of events *r***. The horizontal line indicates the acceptance threshold of 5% error rate.

In our experiments, each dataset is tested using seven methods: GRAPPA-TP (TP), GRAPPA using inversion median (INV), GRAPPA using breakpoint median (BP), MGR, NJ using transposition distances (TP-NJ), NJ using inversion distances (INV-NJ) and NJ using breakpoint distances (BP-NJ). We cannot test our DCJ median here because the scoring procedure of GRAPPA-DCJ generates some median problem instances that are too difficult for it to run. Figure 4 shows the results; we place a line at the 5% error level, the typical threshold of acceptability for accuracy in phylogenetic reconstruction [21].

We make the following two observations.

First, NJ has remarkably good performance when the genomes are close (*r *= 2), but its accuracy quickly drops when the genomes are getting distant, especially when it uses inversion and breakpoint distances. Since NJ is guaranteed to be accurate when the distance between any pair of genomes is very close to the true distance, the good result of TP-NJ also suggests that our distance estimator is valid when the genomes are close.

Second, GRAPPA-TP always returns highly accurate trees, although its performance is slightly worse than TP-NJ for *r *= 2. The accuracy of GRAPPA-TP is also very stable and does not suffer when the genomes are relatively distant. Using breakpoint and inversion median solvers (including MGR) again give very bad results, even for easy datasets of *r *= 2. The results clearly show the importance of model match in genome rearrangement analysis. One should also note that unlike the results in median accuracy, using breakpoint medians in phylogenetic analysis has better performance than using inversion medians. More research in the future is needed to determine the factors contribute to this discrepancy.

Although the number of genomes is relatively small in this test, the high accuracy of GRAPPA-TP makes it ideal as a base method for the DCM-GRAPPA developed by Tang et al. [22], hence can be easily extended to handle several hundred genomes.

## Conclusion

In this paper, we present our new method to handle transpositions and report experimental results on simulated datasets. Although GRAPPA-TP uses a brute-force distance estimator, it remains very accurate for transposition phylogeny. Our studies suggest that model match is very important in both ancestor inference and phylogenetic reconstruction. The main problem of GRAPPA-TP is of course the accuracy and running time of its distance estimator, and a fast and exact method to compute transposition distance is always desirable.

## Methods

We extend GRAPPA to handle transpositions. The new method is named GRAPPA-TP, with two major extensions: a heuristic method to estimate transposition distance, and a new transposition median solver for three genomes.

### Transposition distance estimation

Although no polynomial algorithms for transposition distance has been reported, researchers are able to estimate the distance using the 1.375-approximation by Hartman [10] or the DCJ distance by Yancopoulos et al. [11].

The only existing software that can compute transposition distance is derange2 developed by Blanchette [23], which uses an exhaustively approach to search for a minimum number of transpositions that transform one genome to another. Our tests have shown that when the distance is less than 10% of the number of genes, this method is very fast and the results are very close to the true distances. However, any test above this threshold cannot be finished after several days of computation. For phylogenetic analysis, even when the genomes are close, the distance between some leaves can easily exceed this threshold, thus derange2 will not be applicable. In this paper, we propose a heuristic method which gives satisfactory results.

**Figure 5:**
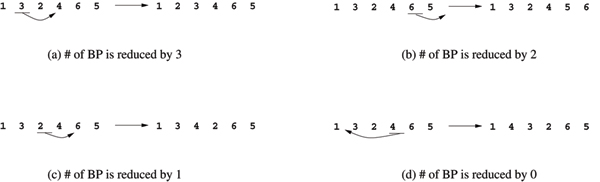
Number of breakpoint changes by applying different transpositions, compared to the identity permutation (1 2 3 4 5 6).

The new distance estimator is based on the following observation: given two genomes *G*_1 _and *G*_2_, a transposition applied on *G*_2 _can reduce the number of breakpoints by 3, 2, 1 or 0, as shown in Figure 5.

This observation suggests that computing the transposition distance can be transferred to find the fewest number of steps that bring the number of breakpoints to zero.

We develop a brute-force method to quickly reduce the number of breakpoints to zero. The algorithm works as follows: it starts from *G*_2 _and moves towards *G*_1_. At each step, it will enumerate all transpositions and apply the one on *G*_2 _that can reduce the most number of breakpoints. It will continue the process until the number of breakpoints becomes 0 (i.e. *G*_2 _is transformed to *G*_1_). The transposition distance is thus the total number of steps used to transform *G*_2 _into *G*_1_. At any given step, it will randomly choose one transposition when there are multiple choices.

The above algorithm is heuristic because in some cases, a transposition at the current step that does not reduce the most number of breakpoints may result in better choices later. Thus, to get more accurate distance, we can repeat the above process several times and report the smallest value as the distance. In our experiments, we found that no more than 10 repeats are needed. This algorithm will always return a distance that is greater or equal to the edit distance.

**Figure 6:**
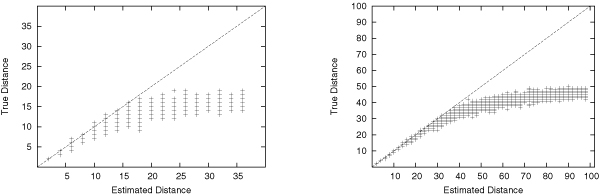
Distance estimation results for 37 genes (left) and 100 genes (right).

Figure 6 shows the performance of our brute-force distance estimation on simulated datasets with 37 and 100 genes. This figure suggests that the estimated distance closely follows the true distance when $\frac{r}{n}$ < 20%, where *r *is the number of transpositions between the two genomes, and *n *is the number of genes. Above this ratio, even our heuristic algorithm (which always returns larger value than the edit distance) will severely under-estimate the true distance. The estimated distance appears to converge onto *n*/2, a ratio close to the conjectured diameter of transposition distances [10].

One should note that this estimator will fail badly for some cases. For example, it only needs four steps to transform the reverse identity genome (7 6 5 4 3 2 1) into (1 2 3 4 5 6 7), while our estimator needs seven steps. However, such cases are very rare, as indicated by Figure 6.

### Transposition median solver

The next step is to develop a transposition median solver to handle the smallest binary trees of three edges. We develop a new median solver that is based on the branch-and-bound method proposed by Siepel and Moret [14].

Given three genomes *G*_1_, *G*_2 _and *G*_3_, and a median genome *G*, we define the *median score *of *G *as the sum of transposition distances from *G *to the three given genomes.

In general, the branch-and-bound approach works as follows:

• Given the three genomes *G*_1_, *G*_2 _and *G*_3_, compute the lower bound on the median score, $D(M)=(d_{G_{1},G_{2}}+d_{G_{2},G_{3}}+d_{G_{3},G_{1}})/2$, where $d_{G_{i},G_{j}}$ denotes the distance between *G*_*i *_and *G*_*j*_.

• Pick one genome as the start and push it into a queue; its median score is the initial best-so-far.

• Iteratively remove a genome *G *from the queue until the queue is empty:

- If the median score of *G *meets the lower bound, $d_{G,G_{1}}+d_{G,G_{2}}+d_{G,G_{3}}=D(M)$, then stop.

- If the median score of *G *is less than the current best-so-far, update the latter.

- create all $\left(\begin{array}{c} n\\ 3 \end{array}\right)/2$ neighboring permutations (one transposition away from *G*), discard those with lower bounds that exceed the best-so-far, and add the surviving ones to the queue.

Clearly, since there are $\left(\begin{array}{c} n\\ 3 \end{array}\right)/2$ neighbors for each step, the success of this algorithm relies on good lower bounds to eliminate as many neighbors as possible. Several lower bounds have been proposed. Among them, the following two bounds are the most effective [14,24]:

(Bound 1) If *G *is a genome on the shortest path from *G*_1 _to the median *M*, then it obeys:

$$d(G_{1},G)+\frac{d(G_{2},G)+d(G_{3},G)+d(G_{2},G_{3})}{2}\leq D(M)$$

(Bound 2) If *G *is a genome on the shortest path from *G*_1 _to the median *M *and *G*' is derived from *G *by applying one inversion, then, if *G*' is also on the shortest path from *G*_1 _to *M*, it obeys:

*d*(*G*_1_, *G*') + *d*(*G*_2_, *G*') + *d*(*G*_3_, *G*') ≤ *d*(*G*_1_, *G*) + *d*(*G*_2_, *G*) + *d*(*G*_3_, *G*) + 1.

In other words, we will ignore those neighbors that can take the search back more than one step.

When the genomes are relatively close, our distance estimation is near optimal, hence the above bounds is still effective. However, these bounds become loose when the genomes are distant, and a new and more effective set of lower bounds should be developed in the future.

The speed of our median solver is regulated by two factors: the distance from the median to its closest leave and the number of genes present. To make the genome length relatively unimportant, we condense the genomes using the concept of conserved adjacency: a gene pair (x, y) is conserved adjacent if (x, y) or its inverse (-y, -x) is present in all genomes as consecutive elements [25]. A block of *k *adjacent genes can be replaced by a pseudo-gene and the total number of genes reduces by *k *- 1 [6]. When the genomes are only several events away, this condensation can easily decrease the genes by 80% and dramatically reduce the number of neighbors being examined at each step.

### Phylogenetic analysis

**Figure 7:**
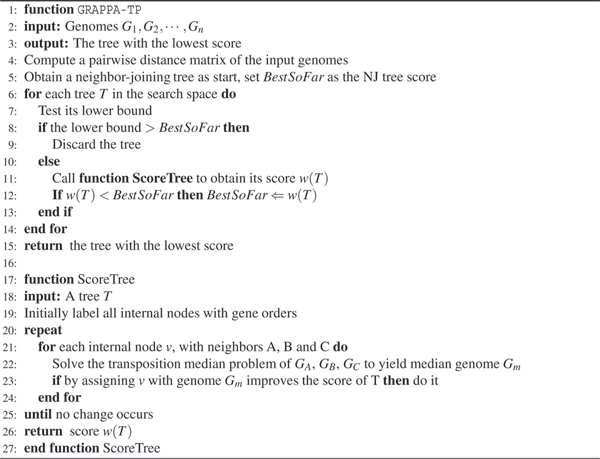
Algorithm overview for GRAPPA-TP.

Computing phylogenies requires two main components for more than three genomes: scoring a given tree, and searching for the best tree based on their scores. The scoring procedure we use is based on the iterative approach implemented in the original GRAPPA, shown as function *ScoreTree *in Figure 7.

The scoring procedure depends on the initial assignment of gene orders to internal nodes, which has no gene-orders assigned when the scoring starts. Internal genomes can be initialized trivially, by giving each internal node a random gene order. However, since the initialization has big impact on the convergence of the scoring procedure, other complex methods are developed and all yield better results. The most used initialization method is the *Nearest Neighbor Method*, which assigns each internal node the median solution from its three nearest leaves, using a median solver of choice. In GRAPPA-TP, we choose to use the transposition median solver in the initialization procedure as well. Although using breakpoint median solver may be faster, it can introduce gene orders with signs that is hard to deal with, due to the fact that transposition does not deal with signs at all.

To search through the large tree space, we will enumerate all trees and use the tightened circular-ordering lower bounds to discard bad trees before scoring them [26]. The lower bound used by GRAPPA is derived from triangular inequalities: let *T *be a tree leaf-labeled by *N *input genomes and *w *be an edge-weighting on *T*; for each pair of leaves *i *and *j*, we have $w_{ij}=\sum_{e\in P_{ij}}w(e)\geq d_{ij}$, where *P*_*ij *_is the path between *i *and *j *in the tree *T*. Set the score $w(T)=\sum_{e\in E(T)}w(e)$. If 1, 2,..., *N *is a circular ordering of the leaves of *T*, then we have 2*w*(*T*) ≥ *d*_1,2 _+ *d*_2,3 _+ ⋯ + *d*_*N*,1_.

This triangular inequality immediately gives us a (circular ordering) lower bound for the tree score, i.e. the tree score *w*(*T*) should at least be $\frac{d_{1,2}+d_{2,3}+\cdots +d_{N,1}}{2}$. In other words, if a tree has lower bound than the best tree score so far, it can be safely discarded because its score will never be smaller the current best. Since our transposition distance computation is not exact, using the lower bound to prune trees become heuristic. However, it performs very well in practice, due to the fact that more than 99.9% trees can be pruned away without being scored. Because the lower bound can be computed very efficiently and is much cheaper than the iterative scoring procedure, such high pruning rate generally indicates more than 100 times speed-up. Other lower bounds have been developed recently, all based on pairwise distances, hence the speed of GRAPPA-TP can be further improved by using those bounds.

## Competing interests

The authors declare that they have no competing interests.

## Authors' contributions

All authors contribute to the development and implementation of the algorithms, and FY and JT are in charge of conducting simulations and analyzing results.
